# Does Margin Length Predict Recurrence After Partial Nephrectomy, or Is Presence Alone Sufficient?

**DOI:** 10.15586/jkc.v13i1.421

**Published:** 2026-04-01

**Authors:** Ural Oğuz, Mehmet Giray Sönmez, Birgül Tok, Pembe Oltulu, Şenol Adanur, Rabia Demirtaş, Mehmet Salih Boğa, Tangül Pınarcı, Gizem Teoman, Sevdegül Aydın Mungan, İlke Onur Kazaz, Gökhan Sönmez, Hülya Akgün, Ertürk Altun, İbrahim Göksoy, Erhan Demirelli, Ercan Öğreden, Doğan Sabri Tok, Salih Al, Eren Baş, Erol Eğrioğlu, Mutlu Ateş, Selçuk Güven

**Affiliations:** 1 Giresun University School of Medicine, Department of Urology, Giresun, Türkiye;; 2 Giresun University Faculty of Arts and Science, Department of Data Science and Analytics, Giresun, Türkiye;; 3 Necmettin Erbakan University School of Medicine, Department of Urology, Konya, Türkiye;; 4 Giresun University School of Medicine, Department of Pathology, Giresun, Türkiye;; 5 Necmettin Erbakan University School of Medicine, Department of Pathology, Konya, Türkiye;; 6 Atatürk University School of Medicine, Department of Urology, Erzurum, Türkiye;; 7 Atatürk University School of Medicine, Department of Pathology, Erzurum, Türkiye;; 8 University of Health Sciences, Antalya Training and Research Hospital, Department of Urology, Antalya, Türkiye;; 9 University of Health Sciences, Antalya Training and Research Hospital, Department of Pathology, Antalya, Türkiye;; 10 Karadeniz Technical University School of Medicine, Department of Pathology, Trabzon, Türkiye;; 11 Karadeniz Technical University School of Medicine, Department of Urology, Trabzon, Türkiye;; 12 Erciyes University School of Medicine, Department of Urology, Kayseri, Türkiye;; 13 Erciyes University School of Medicine, Department of Pathology, Kayseri, Türkiye

**Keywords:** Length of margin, Partial nephrectomy, Recurrence

## Abstract

To evaluate whether the length of positive surgical margin carries a risk for recurrence, data of patients that underwent partial nephrectomy (PN) from six centers were evaluated. Fifty-three patints with positive surgical margins (PSMs) (the PSM group) and 438 patients with negative surgical margin (the NSM group) were included in the present study. Pathologic reevaluations were performed, and surgical margins were measured in micrometers. The number of positive margin areas, and the length of the maximum and total positive margins were evaluated. Data were analyzed using SPSS 27 package program. A p-value less than 0.001 was considered statistically significant. Local recurrence occured in 16.98% of patients in the PSM group and 4.24% of patients in the NSM group. (p<0.001). Patients with PSM were at fourfold increased risk for recurrence. Age, gender, tumor location, tumor side and size, and fuhrman grade were not associated with local recurrence of the tumor (p>0.01). However, positive surgical margin was an important risk factor for local recurrence (p<0.01). No relationship was found between positive margin length and local recurrence (p=0.044). Logistic regression analysis did not identify any parameters associated with local recurrence. The presence of a PSM was significantly associated with an increased risk of local recurrence following PN. The number of positive margin foci and total or maximum length of margin involvement were not associated with recurrence. These findings suggest that it is the presence of PSM, rather than its extent, that may be the primary factor influencing oncological risk.

## Introduction

Partial nephrectomy (PN) is the preferred treatment option for localized renal tumors, particularly T1 lesions, due to its oncological efficacy and renal function preservation. A key component of postoperative evaluation is the assessment of surgical margins. Although a positive surgical margin (PSM) does not always indicate residual tumor or necessitate additional surgical intervention, its presence has been associated with increased risk of local recurrence in some studies (1).

PSMs occur in approximately 2–8% of patients undergoing PN for T1 renal masses (2). Recurrence rates are significantly higher in patients with PSMs, reaching up to 16%, compared to 3% in those with negative surgical margins (3). Despite this known association, there remains limited understanding of whether specific characteristics of PSMs, such as the number of involved foci or the linear extent of margin positivity influence recurrence risk.

To address this gap, the present multicenter retrospective study investigates the association between PSMs and local recurrence after PN, with a particular focus on whether the length of the positive margin serves as a predictive factor. By evaluating quantitative pathological features of margin involvement, we aim to provide further clarity on the prognostic implications of PSMs and inform both surgical decision-making and postoperative surveillance strategies.

## Methods

### Study design

**Figure 1:**
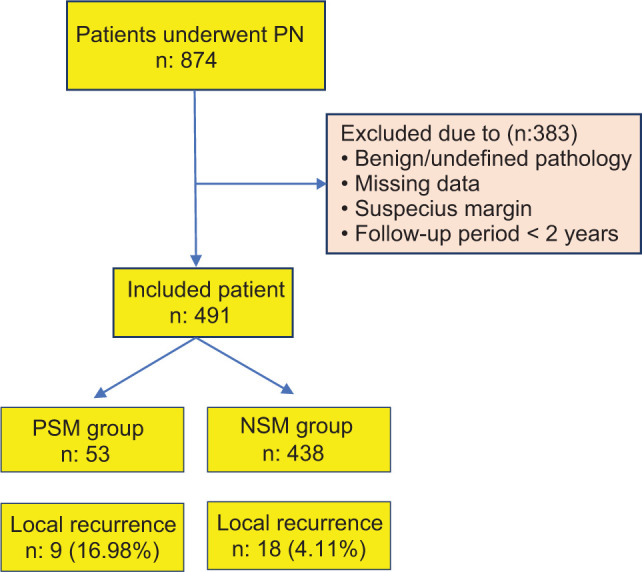
Study flow chart. PSM: Positive surgical margin; NSM: Negative surgical margin.

After obtaining Institutional Ethics Committee approval from Necmettin Erbakan University (ethic number: 2023/4104), a retrospective data analysis was performed by the authors from 6 tertiary hospitals in different regions of Türkiye. Data of 874 patients that underwent PN from 6 centers were evaluated. Patients with benign or undefined pathology, suspicious surgical margins, or incomplete data, as well as those with a follow-up period of less than 2 years, were excluded from the study. Finally, 53 patients with PSMs (the PSM group) and 438 patients with NSM (the NSM group) were included to the present study (Figure 1).

Age, gender, size, side and location of tumor, pathologic subtype, and Fuhrman grade were evaluated. In addition, pathologic reevaluation for all patients were performed, and surgical margins were measured in micrometers by the pathologists. The number of positive margin areas, and the length of the maximum and total positive margins were evaluated. The effect of the length of surgical margin on local recurrence was evaluated in the present study.

### Pathologic evaluation

PN specimens were fixed in 10% buffered formaldehyde solution. Following fixation, the entire specimen was stained with surgical margin dye, and its dimensions and weight were recorded. The specimen was then serially sectioned for macroscopic examination, including assessment of tumor size and gross characteristics. Tissue samples were obtained to include the entire parenchymal surgical margin.

Given the multicenter nature of the study, each participating laboratory processed the samples using their standard protocols and available equipment. After routine tissue processing, paraffin blocks were prepared. Sections with a thickness of 5 micrometers were cut from the paraffin blocks using a microtome and stained with hematoxylin and eosin (H&E) for histopathological evaluation. Microscopic examination was performed using a light microscope, and the length of tumor-positive foci at the surgical margin was measured in micrometers.

For this study, the margin status and measurements were reevaluated by dedicated genitourinary pathologists at each institution. Although a centralized pathology review was not feasible due to the retrospective, multicenter design, participating pathologists adhered to widely accepted histopathological processing principles, including inking of margins and microscopic measurement in micrometers, to enhance consistency across centers.

### Statistical analysis

Data were analyzed using the SPSS 27 package program. The Kolmogorov–Smirnov test first checked the normal distribution of data, and in descriptive statistics, mean ± standard deviation and median (minimum–maximum) were used to express data with and without normal distribution, respectively. Student’s *t*-test was used for normally distributed parameters, and Mann–Whitney U test was used for nonnormally distributed parameters to compare numeric variables. Pearson’s chi-square test or Fisher’s exact test was used to compare the observed frequencies with the expected frequencies. A p-value less than 0.01 was considered statistically significant. Given the relatively small number of recurrence events and multiple comparisons among clinicopathological variables, a more conservative threshold for statistical significance was prespecified as α = 0.01 in order to reduce the risk of type I error.

## Results

Of the 874 patients in the entire cohort who underwent PN, the overall PSM rate was 6.08% (53 out of 874). Following application of the predefined exclusion criteria, including benign or indeterminate histology, suspicious margins, missing variables, and inadequate follow-up, 491 patients were eligible for analysis. Consequently, the proportion of PSMs within the analyzed cohort increased to 10.79% (53 out of 491). The local recurrence rate following PN was 5.49% (n=27), with a median recurrence time of 48 months (IQR=35). The mean follow-up duration was 53.87±36.44 months for the PSM group and 53.59±23.88 months for the NSM group (p=0.157). The male-to-female ratios were 1.4 and 1.9 in the PSM and NSM groups, respectively (p=0.353). The mean ages were 62.43±12.40 years for the PSM group and 59.16±12.32 years for the NSM group (p=0.064). The mean tumor sizes were 3.34±1.44 cm for the PSM group and 3.69±1.78 cm for the NSM group (p=0.165) (Table 1).

**Table 1:** Baseline patient, disease, and operation characteristics.

		PSM group N=53	NSM group N=438	p
**Age at Surgery – Mean ± SD**		62.43±12.40	59.16±12.32	0.064^a^
**Gender**	Female	22 (12.9%)	149 (87.1%)	0.353^b^
	Male	31 (9.7%)	289 (90.3%)	
**Tumor Size (cm)**		3.34±1.44	3.69±1.78	0.165^a^
**Fuhrman Grade**		2.00±0.83	1.90±0.68	
**Fuhrman – Noninformed**		9 (13%)	60 (87%)	0.302^b^
**Fuhrman – 1 or 2**		316 (90.5%)	33 (9.5%)	
**Fuhrman – 3 or 4**		62 (84.99%)	11 (15.1%)	
**Histological Type – Clear cell**		23 (11.10%)	185 (88.90%)	0.989^b^
**Histological Type – Other**		30 (10.6%)	253 (89.4%)	
**Follow Up (Month)**		51.60±36.44	-	-
**Local Recurrence (+)**		9 (39.1%)	14 (60.9%)	0.000^b^
**Local Recurrence (-)**		44 (9.6%)	413 (90.4%)	
**Polar Localization – Upper**		19 (15.6%)	103 (84.4%)	0.137^b^
**Polar Localization – Middle**		16 (9.9%)	146 (90.1%)	
**Polar Localization – Lower**		18 (8.7%)	189 (91.3%)	
**Tumor Side – Left**		26 (10.8%)	215 (89.2%)	0.997^b^
**Tumor Side – Right**		27 (10.8%)	223 (89.2%)	
**Renal Score**		5.15±1.89	5.50±1.56	0.060^a^
**Resection Technique – Enucleation**		17 (15.9%)	90 (84.1%)	0.161^b^
**Resection Technique – Enucleoresection**		1 (9.1%)	10 (90.9%)	
**Resection Technique – Standard Resection**		35 (9.4%)	337 (90.6%)	
**EBL-mL**		59.25±110.412	91.38±144.82	0.093^a^
**Operation Technique – Open**		28 (11.7%)	212 (88.3%)	0.064^b^
**Operation Technique – Laparoscopic**		17 (15.00%)	96 (85.00%)	
**Operation Technique – Robotic**		8 (5.9%)	127 (94.1%)	

a Mann–Whithey U test; bChi square test.

PSM: Positive surgical margin; NSM: Negative surgical margin; EBL: Estimated blood loss.

Pathological high-grade tumors (Fuhrman grade III or IV) were identified in 20.7% of the PSM group and 14.15% of the NSM group. Although the incidence of high-grade tumors was higher in the PSM group, it was not statistically significant (p=0.302). Local recurrence occurred in 16.98% of patients in the PSM group compared to 4.11% in the NSM group (p<0.001). Patients with PSM had a fourfold increased risk of recurrence according to the odds ratio from logistic regression analysis.

Positive margins were detected in a single area in 31 (58.5%) patients and in multiple areas in 22 (41.5%) patients. The median total length of the surgical margin in the PSM group was 4340 micrometers (IQR=9697). Among the PSM group, the median total length of the surgical margin was 1700 micrometers (IQR=6400) in patients with local recurrence (n=9) and 5237.5 micrometers (IQR=10738) in patients without local recurrence (n=44) (p=0.044). However, this value did not reach the predefined level of statistical significance (α = 0.01) and was therefore interpreted as a nonsignificant trend (Table 2). Moreover, no consistent gradient suggesting increasing recurrence risk with increasing margin length was observed.

**Table 2:** Association between surgical margin and local recurrence in patients with positive surgical margins.

	Local recurrence (+)		Local recurrence (–)		p-value	Mann–Whitney U statistics
	Median	IQR	Median	IQR		
Number of involved foci	1	2	1	1	0,917	193
Total length of the positive margin	1700	6400	5237,5	10738	0,044	113
Maximum length of the positive margin	950	5150	4575,5	9038	0,017	98

Our results indicate that age, gender, tumor location, tumor side, size, and Fuhrman grade were not associated with local recurrence of the tumor (p>0.01). However, positive surgical margins were found to be an important risk factor for local recurrence (p<0.001) (Table 1). The number of positive margin areas and the length of the maximum and total positive margins were not associated with local recurrence. According to our data, no relationship was found between positive margin length and local recurrence (p=0.044, α= 0.01). Furthermore, logistic regression analysis did not identify any parameters associated with local recurrence.

## Discussion

PN has become a standard treatment modality especially for T1 renal tumors due to better preservation of renal function and decreasing the risk for cardiovascular disorders (4, 5). The main concern of the nephron sparing surgery is the inability to achieve NSMs. Local recurrence may not only be attributable to local spread of the tumor by microvascular embolization, or true multifocality, but also to incomplete resection of the primary tumour (6). It is well known that positive surgical margin is associated with increased risk for recurrence. The risk of local recurrence increases from 3% to 16% in patients with PSMs (3). However, the more nuanced question, “which specific characteristics of positive surgical margins contribute most to recurrence?,” remains largely unanswered in the existing literature. Thus, the present study aimed to explore this further by examining the length and number of positive surgical margins and their impact on local recurrence.

The majority of recurrences tend to happen within the first 2 years following surgery. It has been demonstrated that local recurrences were mostly detected within the first 2–5 years following surgery (7). Therefore, we included patients with at least 2 years of follow-up reports in the present study.

In the light of our data, PSMs are the most important risk factors for local recurrence. However, a relationship was not found between the length of positive margin and local recurrences. Therefore, the absence of a robust and biologically plausible dose–response relationship further supports the interpretation that margin length alone should not be used as a determinant of recurrence risk. On the other hand, Fuhrman grade and tumor size were not statistically associated with local recurrence.

Anatomic complexity may affect the outcomes of PN. However, studies mostly did not evaluate whether a nephrometry-score (PADUA or RENAL) was associated with local recurrence. Matos et al. revealed that RENAL score was not related to PSM, which is similar to our data (8).

Effect of surgical techniques such as enucleation, enucleoresection, or resection have also been investigated as to whether they have any impact on surgical margins. The only prospective data published by Minervini et al. showed that resection technique significantly impacts PSM rates (9). However, in recent meta-analysis, it has been revealed that, similar to our study, surgical technique (simple enucleation versus standard PN) has no impact on surgical margin and local recurrence (10, 11).

It is well established that tumor size and histologic Fuhrman grade are important prognostic factors for kidney cancers (12). European Association of Urology RCC Guidelines suggest using the Leibovich model for clear cell RCC to evaluate risk for recurrence and to propose a follow-up schedule (13, 14). Fuhrman grade and tumor size are both important risk factors for recurrence after PN, as suggested in Leibovich model (14, 15). However, it was also revealed in the literatüre that they were not related to PSMs and local recurrence as it is in the present study (16). It was also investigated in the literature that the pathologic upstaging to T3a after PN is another important risk factor for local recurrence (17, 18). However, due to the low number of patients with pT3a tumor in our series, we could not evaluate the effect of upstaging to T3a on local recurrence.

Interestingly, univariable analysis revealed a trend toward shorter margin lengths in patients who experienced recurrence. However, this observation should be interpreted with extreme caution. Firstly, the study included a limited number of recurrence events, increasing the susceptibility to random variation. Secondly, the tumor’s biological behavior and mere violation of tumor boundaries may be more important than the measured linear extent of involvement. For these reasons, and in accordance with our prespecified conservative significance threshold (α = 0.01), we did not interpret p = 0.044 as evidence of a true association.

Although the pathologic reevaluation was performed according to the same standards in all centers, the retrospective nature of the study could be considered as a limitation of this study. Despite this shortcoming, this is a unique article investigating the relationship between tumor length in the positive margin and local recurrence. We believe that the present study may contribute to the improvement of the writing guidelines of pathology reports especially by supporting with further postoperative studies with larger series.

## Conclusion

In this multicenter study, the presence of a PSM was significantly associated with an increased risk of local recurrence following PN. However, the number of positive margin foci, as well as the total or maximum length of margin involvement, did not show a statistically significant association with recurrence in the multivariable analysis. These findings suggest that it is the presence of PSM, rather than its extent, that may be the primary factor influencing oncological risk. Further prospective studies with larger cohorts are needed to determine whether the characteristics of margins should impact postoperative surveillance or treatment strategies.

## Mandatory Disclosure on Use of Artificial Intelligence

The authors declare that AI-assisted tools (ChatGPT or Gemini) were used only for minor language editing during the revision process. No AI tools were used in the drafting of the manuscript. All references have been manually verified for accuracy and relevance.

## Author Contributions

All authors contributed equally to this article.

## References

[1] Sundaram V, Figenshau RS, Roytman TM, Kibel AS, Grubb RL, Bullock A, et al. Positive margin during partial nephrectomy: does cancer remain in the renal remnant? Urology. 2011 Jun;77(6):1400–3. 10.1016/j.urology.2010.12.016 21411126

[2] Volpe A, Capitanio U, Falsaperla M, Giannarini G, Palumbo C, Antonelli A, et al. Nephrectomy for renal tumors: recommendations of the Italian Society of Urology RCC working group. Minerva Urology and Nephrology. 2024 Feb;76(1):9–21. 10.23736/S2724-6051.24.05772-0 38426419

[3] Wood EL, Adibi M, Qiao W, Brandt J, Zhang M, Tamboli P, et al. Local tumor bed recurrence following partial nephrectomy in patients with small renal masses. Journal of Urology. 2018 Feb;199(2):393–400. 10.1016/j.juro.2017.09.072 28941919

[4] Capitanio U, Terrone C, Antonelli A, Minervini A, Volpe A, Furlan M, et al. Nephron-sparing techniques independently decrease the risk of cardiovascular events relative to radical nephrectomy in patients with a T1a-T1b renal mass and normal preoperative renal function. European Urology. 2015 Apr;67(4):683–9. 10.1016/j.eururo.2014.09.027 25282367

[5] Scosyrev E, Messing EM, Sylvester R, Campbell S, Van Poppel H. Renal function after nephron-sparing surgery versus radical nephrectomy: results from EORTC randomized trial 30904. European Urology. 2014 Feb;65(2):372–7. 10.1016/j.eururo.2013.06.044 23850254

[6] Lee Z, Jegede OA, Haas NB, Pins MR, Messing EM, Manola J, et al. Local recurrence following resection of intermediate-high risk nonmetastatic renal cell carcinoma: an anatomical classification and analysis of the ASSURE (ECOG-ACRIN E2805) adjuvant trial. Journal of Urology. 2020 Apr;203(4):684–689. 10.1097/JU.0000000000000588 31596672 PMC7337326

[7] Chin AI, Lam JS, Figlin RA, Belldegrun AS. Surveillance strategies for renal cell carcinoma patients following nephrectomy. Reviews in Urology. 2006 Winter;8(1):1–7. 16985554 PMC1471767

[8] Matos AC, Dall’Oglio MF, Colombo JR, Crippa A, Juveniz JAQ, Argolo FC. Predicting outcomes in partial nephrectomy: is the renal score useful? International Brazilian Journal of Urology. 2017 May–Jun;43(3):422–431. 10.1590/s1677-5538.ibju.2016.0315 28266814 PMC5462132

[9] Minervini A, Campi R, Lane BR, Cobelli OD, Sanguedolce F, Hatzichristodoulou G, et al. Impact of resection technique on perioperative outcomes and surgical margins after partial nephrectomy for localized renal masses: a prospective multicenter study. Journal of Urology. 2020 Mar;203(3):496–504. 10.1097/JU.0000000000000591 31609167

[10] Minervini A, Campi R, Sessa F, Derweesh I, Kaouk JH, Mari A, et al. Positive surgical margins and local recurrence after simple enucleation and standard partial nephrectomy for malignant renal tumors: systematic review of the literature and meta-analysis of prevalence. Minerva Urologica e Nefrologica. 2017 Dec;69(6):523–538. 10.23736/S0393-2249.17.02864-8 28124871

[11] Bertolo R, Pecoraro A, Carbonara U, Amparore D, Diana P, Muselaers S, et al. Resection techniques during robotic partial nephrectomy: a systematic review. European Urology Open Science. 2023 Apr 29:52:7–21. 10.1016/j.euros.2023.03.008 PMC10172691 37182118

[12] Sun M, Shariat SH, Cheng C, Ficarra V, Murai M, Oudard S, et al. Prognostic factors and predictive models in renal cell carcinoma: a contemporary review. European Urology. 2011 Oct;60(4):644–61. 10.1016/j.eururo.2011.06.041 21741163

[13] van den Brink L, Reijerink MAA, Henderickx M M E L, Bex A, Jamaludin FS, Beerlage HP, et al. Is frequent imaging necessary? Impact of computed tomography during follow-up after surgical treatment for nonmetastatic renal cell carcinoma: a systematic review. European Urology Oncology. 2025 Jun 8(3):829–840. 10.1016/j.euo.2024.11.014 39665918

[14] Leibovich MC, Blute ML, Cheville JC, Lohse CM, Frank I, Kwon ED, et al. Prediction of progression after radical nephrectomy for patients with clear cell renal cell carcinoma: a stratification tool for prospective clinical trials. Cancer. 2003 Apr 1;97(7):1663–71. 10.1002/cncr.11234 12655523

[15] Tabourin T, Pinar U, Parra J, Vaessen C, Bensalah CK, Audenet F, et al. Impact of renal cell carcinoma histological variants on recurrence after partial nephrectomy: a multi-institutional, prospective study (UROCCR Study 82). Annals of Surgical Oncology. 2022 Oct;29(11):7218–7228. 10.1245/s10434-022-12052-8 35780452

[16] Demirel HC, Çakmak S, Yavuzsan AH, Yeşildal C, Türk S, Dalkılınç A, et al. Prognostic factors for surgical margin status and recurrence in partial nephrectomy. International Journal of Clinical Practice. 2020 Oct;74(10):e13587. 10.1111/ijcp.13587 32558097

[17] Shah PH, Moreira DM, Patel VR, Gaunay G, George AK, Alom M, et al. Partial nephrectomy is associated with higher risk of relapse compared with radical nephrectomy for clinical stage T1 renal cell carcinoma pathologically up staged to T3a. 2017 Aug;198(2):289–296. 10.1016/j.juro.2017.03.012 28274620

[18] Takagi T, Yoshida K, Wada A, Kondo T, Fukuda, Ishihara H, et al. Predictive factors for recurrence after partial nephrectomy for clinical T1 renal cell carcinoma: a retrospective study of 1227 cases from a single institution. International Journal of Clinical Oncology. 2020 May;25(5):892–898. 10.1007/s10147-020-01632-x 32048086

